# Ambiguity in Communicating Intensity of Physical Activity: Survey Study

**DOI:** 10.2196/16303

**Published:** 2020-05-28

**Authors:** Hyeoneui Kim, Jaemin Kim, Ricky Taira

**Affiliations:** 1 School of Nursing Duke University Durham, NC United States; 2 Cancer Care Ontario Toronto, ON Canada; 3 Department of Radiological Science University of California Los Angeles Los Angeles, CA United States

**Keywords:** exercise, health communication, exercise intensity

## Abstract

**Background:**

Communicating physical activity information with sufficient details, such as activity type, frequency, duration, and intensity, is vital to accurately delineate the attributes of physical activity that bring positive health impact. Unlike frequency and duration, intensity is a subjective concept that can be interpreted differently by people depending on demographics, health status, physical fitness, and exercise habits. However, activity intensity is often communicated using general degree modifiers, degree of physical exertion, and physical activity examples, which are the expressions that people may interpret differently. Lack of clarity in communicating the intensity level of physical activity is a potential barrier to an accurate assessment of exercise effect and effective imparting of exercise recommendations.

**Objective:**

This study aimed to assess the variations in people’s perceptions and interpretations of commonly used intensity descriptions of physical activities and to identify factors that may contribute to these variations.

**Methods:**

A Web-based survey with a 25-item questionnaire was conducted using Amazon Mechanical Turk, targeting adults residing in the United States. The questionnaire included questions on participants’ demographics, exercise habits, overall perceived health status, and perceived intensity of 10 physical activity examples. The survey responses were analyzed using the R statistical package.

**Results:**

The analyses included 498 responses. The majority of respondents were females (276/498, 55.4%) and whites (399/498, 79.9%). Numeric ratings of physical exertion after exercise were relatively well associated with the 3 general degree descriptors of exercise intensity: light, moderate, and vigorous. However, there was no clear association between the intensity expressed with those degree descriptors and the degree of physical exertion the participants reported to have experienced after exercise. Intensity ratings of various examples of physical activity differed significantly according to respondents’ characteristics. Regression analyses showed that those who reported good health or considered regular exercise was important for their health tended to rate the intensity levels of the activity examples significantly higher than their counterparts. The respondents’ age and race (white vs nonwhite) were not significant predictors of the intensity rating.

**Conclusions:**

This survey showed significant variations in how people perceive and interpret the intensity levels of physical activities described with general severity modifiers, degrees of physical exertion, and physical activity examples. Considering that these are among the most widely used methods of communicating physical activity intensity in current practice, a possible miscommunication in assessing and promoting physical activity seems to be a real concern. We need to adopt a method that represents activity intensity in a quantifiable manner to avoid unintended miscommunication.

## Introduction

### Importance of Capturing Information on Physical Activity Intensity

Being physically active is essential for maintaining good health [1]. Assessment, intervention, and outcome evaluation related to one’s health status now require incorporating a patient’s lifestyle information [2-5]. To facilitate the use of lifestyle information in patient care, the Office of National Coordinator for Health Information Technology (ONC) and the National Academy of Medicine (NAM; formerly known as the Institute of Medicine) recognized the 9 social and behavioral health domains, including physical activity, that need to be incorporated into electronic health records (EHRs) in a structured format [6,7].

Undoubtedly, regular and sufficient physical activity is among the most essential lifestyle approaches for staying healthy. Health care professionals often prescribe physical activity as part of a treatment regimen for a patient to facilitate recovery from a disease or to prevent further aggravation of the disease. Ascertaining whether a person is getting a sufficient level of physical activity requires examining 4 attributes characterizing physical activity, including frequency, intensity, time (ie, duration), and type (FITT) of the activity [8]. Specifying activity FITT is also essential when recommending physical activity to a patient, as it helps patients understand what constitutes an adequate level of physical activity that can have a positive impact on their health.

It is relatively straightforward to describe the frequency, time, and types of physical activity, as there are agreed-upon methods of objectively representing these data types. However, communicating the notion of intensity can be challenging because of its subjective nature and dependence on individual biases and internal calibrations.

### Quantifying Physical Activity Intensity

There are several methods for quantifying the intensity level of a physical activity. The metabolic equivalent of task (MET) value is a measure of energy expenditure required to perform a task relative to the energy expenditure of an average person seated at rest. Thus, if an activity has an MET value of 2, this translates to an activity intensity that requires twice the energy of the resting reference event [9,10]. The MET value thus provides a relatively standardized means of describing the intensity level of a particular physical activity for healthy adults [11]. In general, we classify activities with a MET value less than 3 as light-intensity activities, those between 3 and 6 as moderate-intensity activities, and those greater than 6 as vigorous-intensity activities. Individuals may require a different amount of energy to complete the same task depending on the person’s age, BMI, and overall physical fitness. The corrected MET value is a weighted MET value calculated incorporating such dependencies [12].

The maximum oxygen uptake rate (%VO_2_ max) refers to the relative amount of oxygen a person uses during physical activity. Slightly different range values may apply for the intensity categories for %VO_2_max depending on gender and age. For example, the classification of *vigorous* for an adult female aged between 18 and 40 years has a %VO_2_ max range of 64% to 91% [13]. In other words, if a young adult female is consuming 64% to 91% of her %VO_2_ to perform an activity, she is involved in an activity with vigorous intensity.

Finally, maximum heart rate (%HR max) is another widely used method for quantifying activity intensity [14]. In general, when using this metric, an activity is vigorous if it causes the heart rate (HR) of the person performing the activity to increase to 76% to 96% of his or her %HR max.

Although these metrics allow one to quantify the intensity level of a physical activity event, they are often not practical to obtain because they require specialized instruments and calculations. Therefore, they are not widely used to describe activity intensity in normal communications with patients. In addition, these measures are not free from limitations, and numerous studies suggested revisiting the reliability and validity of these measures [14-16].

Various qualitative characterizations have also been defined to assess a patient’s activity intensity. The Rating of Perceived Exertion (RPE) scale represents a person’s self-reported exertion level after a particular activity. The Borg scale, the most widely used RPE, rates a perceived exertion level from a value of 6, which indicates no exertion at all, to a value of 20, which indicates maximum exertion [8,17]. The Borg scale is easy to implement and is considered sufficiently accurate for many purposes [18]. However, studies also reported limitations of this scale, for example, underestimating activity intensity compared with what is reflected in exercise HRs [19,20].

The talk test is another simple method of describing the intensity level of physical activity that a person perceives. The talk test is based on the extent to which a person can verbally respond in a conversation during the exerted activity [21]. For example, if a person is unable to converse during physical activity, he or she is considered engaged in a vigorous-intensity activity. However, similar to the other intensity assessment methods described earlier, studies have reported mixed findings on the validity of the talk test as a clinical tool for assessing activity intensity [8,22,23].

### Limitations in the Daily Communication of Physical Activity Intensity

As described earlier, a number of efforts have been put forward to devise means for characterizing physical activity intensity, although none are free from the aforementioned limitations. The granularity and levels of agreement among these measures can be quite variable. In addition, the real-world constraints associated with the application of these measures in the clinical setting are important to consider. In most everyday communication with patients, inquiry of activity intensity is presented to the patient using everyday natural language expressions. Compared with calibrated measures, the response from these types of inquiries can have widely varied interpretations.

As shown in Table 1, general degree descriptors, such as light, moderate, and vigorous, are among the most frequently used methods for describing activity intensity. Many questionnaires and scales that assess people’s physical activity level also add additional descriptors to express activity intensity. Many of them include the exertion levels expressed with a degree of increment in sweating, HR, and breathing after exercise to denote activity intensity. In addition, specific activity types are often accompanied by appropriate performance descriptors (eg, fast and for pleasure) to provide additional specificity.

**Table 1:** Activity intensity descriptions used in various physical activity questionnaires.

Questionnaire	Intensity description examples
California Health Interview Survey 2009 Adult Questionnaire	Think about *vigorous activities* you did in your free time that *take hard physical effort*, such as *aerobics, running, soccer, fast bicycling, or fast swimming*. Again, do not include walking. During the last 7 days, did you do any *vigorous physical activities* in your free time?
Neighborhood Physical Activity Questionnaire	In a usual week, how many times do you do *moderate-intensity* leisure-time physical activities that *do not make you breathe harder or puff and pant*?
National Health and Nutrition Examination Survey Physical Activity and Physical Fitness Physical Activity Questionnaire (version 1998)	Moderate activity: Over the past 30 days, did you do *moderate activities* for at least 10 min that *caused light sweating*? (*brisk walking or bicycling for pleasure*)
Health-enhancing physical activity and Office in Motion Questionnaire	Think about *moderate physical activities* that *make you breathe somewhat harder* and may include *continuous walking, hiking, dancing, gardening, or sport activities*. Currently, do you do any physical activities that *make you breathe somewhat harder*?

In 2014, the ONC and the NAM [6,7] proposed recommendations to document a patient’s physical activity information in the EHR using the following 2 questions from *Exercise Vital Signs* [24]: (1) *On average, how many days per week do you engage in moderate to strenuous exercise (such as walking fast, running, jogging, dancing, swimming, biking, or other activities that cause a light or heavy sweat)?* and (2) *On average, how many minutes do you engage in exercise at this level?*

These 2 questions are assumed to capture the minimum necessary information related to a patient’s overall exercise habits, including frequency, intensity, and duration. However, the intensity information related to the first question might not adequately reflect individual patients’ exercise level and potentially hamper a clinician’s effort to make an accurate assessment and/or provide an effective recommendation for physical activity as part of a treatment regimen.

In summary, the intensity characterization of physical activity is an essential component when assessing and recommending a physical activity. There exists a risk of missing vital details when the intensity of physical activity is expressed using general descriptors that do not incorporate individual differences in intensity experience and/or perception. As a first step to identify potential gaps in communicating activity intensity, we investigated how people perceive or interpret the intensity levels when described with general degree modifiers, physical exertion descriptions, and activity scenarios.

### Study Aims

We conducted a survey to assess how people perceive the intensity levels of physical activity expressed by methods commonly used to inquire exercise intensity in daily communication. In particular, we aimed to answer the following research questions: (1) How different or similar are people’s perception of physical activity intensity with respect to the use of general degree modifiers, degree of physical exertion, and activity examples? and (2) Are there any patterns or associations between people’s characteristics and perception of the presented intensity descriptions?

## Methods

### Survey Questionnaire

We designed a questionnaire survey to collect 3 types of information: (1) survey participants’ demographics, which included age, gender, race, and ethnicity; (2) participants’ exercise habits, including frequency, duration, and intensity (this information category also included each respondent’s overall health status and attitude toward regular exercise); and (3) the perceived intensity levels of different physical activity examples. Survey participants were asked to rate the intensity levels of 10 everyday physical activities using numeric scores ranging from 0 to 10, where 0 indicates activity causes no exertion and 10 indicates activity causes extreme exertion. We selected the 10 activity examples used in the 2011 Physical Activity Compendium [9]. Survey participants had the option to mark “don’t know” if they were unfamiliar with the presented activity. The survey questionnaire is included as a supplemental file (Multimedia Appendix 1).

### Recruitment

We recruited survey participants using the Web-based framework provided by Amazon Mechanical Turk (MTurk). MTurk is a crowdsourcing marketplace where various tasks are outsourced to a distributed workforce who can perform these tasks virtually [25,26]. Tasks completed using MTurk vary from conducting simple data validation to more subjective tasks such as survey participation. This study was exempted by the institutional review board. We limited participation to adults residing in the United States.

### Statistical Analysis

We descriptively analyzed the participants’ demographics, exercise habits, and perceived intensity of different activity types. We also examined whether there were any significant associations between participants’ characteristics and their intensity perception. This survey asked people’s subjective perception and experience of the intensity of physical activity, where no gold standard answer exists. Therefore, the analysis focused on examining how similar or dissimilar people’s perceptions of physical activity intensity were. All data analyses were performed using the R statistical package, version 3.5.1 [27].

#### Power

To be able to generalize the survey results to the general adult population living in the United States with a 95% CI and a 5% margin of error, we estimated a sample size of at least 385 responses. We received a total of 522 responses.

#### Data Exclusion

After removing 24 unreliable responses (eg, too many unanswered questions or implausible answers), there remained 498 responses for analyses.

## Results

### Survey Participants’ Characteristics

The participants’ age, race, and sex statistics are summarized in Table 2. The majority of the survey participants were white (399/498, 79.9%), and there were more females (276/498, 55.4%) than males. The mean age of the respondents was 40.59 (SD 12.56) years. The majority of the participants reported that they were in good (290/498, 58.23%) or excellent (43/498, 8.63%) health and considered regular exercise as being very (184/498, 36.95%) or extremely important (108/498, 21.69%) for their health.

Approximately 72.6% (362/498) of the respondents answered that they exercised regularly. The frequency, duration, and intensity level reported by the majority of these 362 regular exercisers were 3 to 4 days a week (n=162), for 30 to 60 min (n=228), at a moderate intensity level (n=227). Among the 136 people who responded that they did not exercise regularly, 93 answered exercising occasionally. The majority of these 93 sporadic exercisers indicated that they exercised, on average, about 1 or 2 days per week (n=85), for less than 30 min (n=63), at a mild intensity level (n=69). The remaining 43 people responded that they were not exercising at all.

The proportions of regular exercisers differed according to the respondent’s characteristics, as shown in Table 3. The significance of the differences was tested using the two-sample proportion test and the chi-square test. Men, people without a known medical condition that limits their physical activity, those using an activity tracker, those considering themselves to be in good health, and those thinking that regular exercise is important for their health were more likely to exercise regularly. There was no significant difference in the age distributions between regular exercisers and nonregular exercisers (*P*=.82) when tested with Student *t* test.

**Table 2:** Age, sex, and race distributions of the survey participants.

Race	Age (years), mean (SD)	Number of respondents by sex and race, n (%)		
		Female (n=276)	Male (n=221)	Other (n=1)
American Indian or Alaska Native	40 (5.66)	1 (0.36)	1 (0.45)	0 (0)
Asian	34 (8.92)	12 (4.3)	13 (5.8)	0 (0)
Black or African American	37 (12.38)	23 (8.3)	15 (6.7)	0 (0)
Hispanic or Latino	32 (10.67)	9 (3.2)	16 (7.2)	0 (0)
Native Hawaiian or Other Pacific Islander	52 (NA)	0 (0.0)	1 (0.45)	0 (0)
Other	41 (12.68)	6 (2.1)	2 (0.90)	0 (0)
White	39 (8.23)	225 (81.5)	173 (78.2)	1 (100)
Total	40.59 (12.56)	276 (100)	221 (100)	1 (100)

**Table 3:** Regular exercise ratios by participants’ characteristics.

Participants’ characteristics		Regular exerciser, n (%)	*P* value
**Sex**			**.002**
	Female	185 (67.0)	
	Male	177 (80.1)	
**Using activity tracker**			**<.001**
	Yes	134 (86.5)	
	No	228 (66.5)	
**Race**			**.49**
	American Indian or Alaska Native	1 (50)	
	Asian	18 (72)	
	Black or African American	31 (82)	
	Hispanic or Latino	17 (68)	
	Native Hawaiian or Other Pacific Islander	1 (100)	
	Other	4 (50)	
	White	290 (72.7)	
**Perceived health status**			**<.001**
	Excellent	41 (95)	
	Good	229 (78.9)	
	Fair	75 (54)	
	Poor	17 (68)	
**Age (years)**			**.82**
	≥65	14 (78)	
	<65	348 (72.5)	
**Importance of regular exercise**			**<.00**
	Extremely important	103 (95.4)	
	Very important	160 (86.9)	
	Moderately important	89 (65)	
	Somewhat important	9 (16)	
	Not at all	1 (8)	
**Having a medical condition limiting physical activity**			**<.001**
	Yes	63 (64)	
	No	293 (75.7)	

### Perceived Exercise Intensity

**Figure 1:**
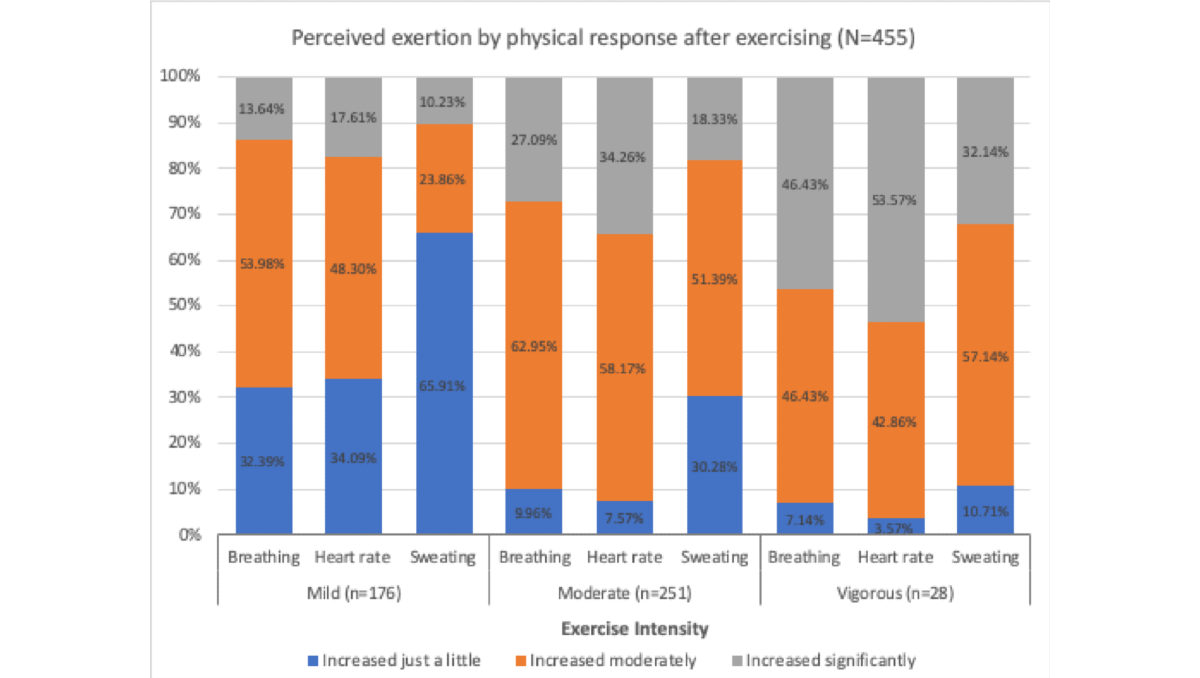
Level of physical exertion reported for the different intensities of exercise.

We asked 455 participants who exercised regularly (n=362) or sporadically (n=93) to describe the intensity of the exercise they usually performed in 2 ways: (1) using general degree modifiers and (2) based on the physical exertion they experienced after the exercise. As a means of describing the level of exertion, we presented 3 types of physiologic responses: an increment in HR, breathing rate, and sweating. Note that these physiologic responses are also commonly used to describe intensity levels in various validated physical activity questionnaires. Figure 1 shows that the exertion levels reported by the participants after exercise were not always linearly related to the exercise intensity they described using general degree modifiers. For example, a few respondents reported only a minor increase in breathing, sweating, and HR after vigorous exercise. Similarly, some people reported experiencing a significant increase in these physiologic parameters after mild-intensity exercise.

**Figure 2:**
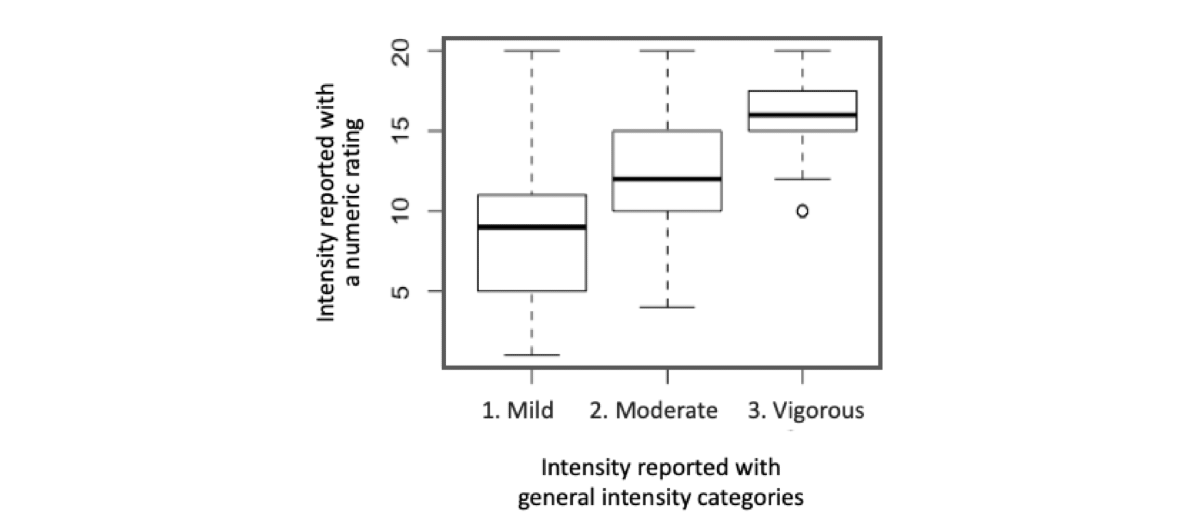
The distribution of numeric intensity rating reported for the general intensity categories.

The participants who exercised regularly or sporadically (n=455) were also asked to rate the perceived intensity level of the exercise they usually performed using 3 general intensity levels and a 20-point scale, where 0 indicates no exertion at all and 20 indicates extreme exertion. Figure 2 shows how the numeric ratings of perceived exertion are distributed in the 3 intensity levels. Although there were small overlaps, the distributions of the ratings were well differentiated among the 3 intensity levels. The average numeric intensity ratings were significantly different among the 3 intensity levels when tested with the Kruskal-Wallis test (*P*<.001).

### Perceived Intensity Levels of 10 Physical Activity Examples

**Figure 3:**
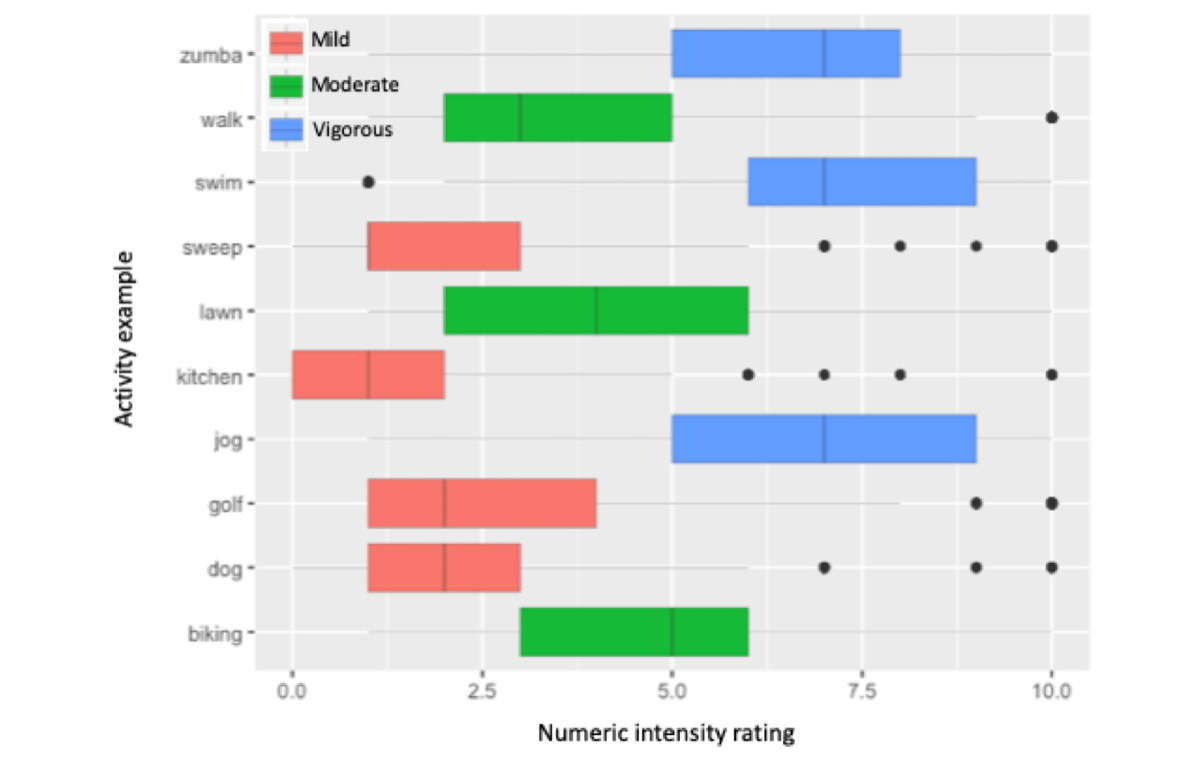
Numeric intensity ratings assigned to 10 activity examples (zumba: aerobic dancing such as Zumba; walk: walking at a pace of 3.5 miles per hour; swim: fast lap swimming–freestyle; sweep: sweeping garage, sidewalk, or outside of the house; lawn: lawn mowing with a hand mower; kitchen: kitchen activities such as cooking, washing dishes, and cleaning up; jog: jogging at a pace of 5 to 7 miles per hour; golf: golf—walking and carrying clubs; dog: walking a dog; and biking: biking at a park).

Figure 3 shows the overall distribution of the intensity ratings that the participants assigned to the 10 activity examples. Although there exist substantial variations in the ratings of all 10 activities, the participants tended to perceive aerobic dancing, fast lap swimming, and jogging as more intense, whereas they considered kitchen works, walking the dog, and sweeping a driveway as less intense.

The 10 activity examples formed approximately 3 intensity groups as color coded in Figure 3. As a reference to standardized intensity information, we included in parentheses below the corresponding MET values proposed in the study by Ainsworth et al [9] for each activity. The activities that received relatively high-intensity ratings included *jogging at a pace of 5 to 7 miles per hour* (MET 8.3-11), *fast lap swimming–freestyle* (MET 9.8), and *aerobic dancing such as Zumba* (MET >5.0). The activities with middle range intensity ratings are *biking at a park* (MET 4.0), *lawn mowing with a hand mower* (MET 6.0), and *walking at a pace of 3.5 miles per hour* (MET 4.3). *Walking a dog* (MET 3.0); *golf—walking and carrying clubs* (MET 4.3); *kitchen activities such as cooking, washing dishes,* and *cleaning up* (MET 3.3); and *sweeping garage, sidewalk, or outside of the house* (MET 4.0) received relatively low-intensity ratings from the participants.

### Factors Associated With the Differences in Perceived Intensity Ratings

We conducted regression analyses to examine how the participants’ characteristics affect the perceived intensity ratings of the 10 activity examples. We dichotomized race (white vs nonwhite), perceived health status (good/excellent vs fair/poor), and importance of exercise (moderately/very/extremely vs slightly/not at all). We also created 3 additional characteristics that reflect the relative exertion level experienced by the participants by comparing the participants’ self-reported physical exertion level with the self-reported exercise intensity. The respondents were classified as *less increase in HR or respiratory rate* or *sweat less* if they reported a lesser level of exertion than the level of the exercise intensity they performed. For example, we classified a respondent to a *lesser increase in HR* group when she reported experiencing a small increase in HR after performing a moderate- or vigorous-intensity exercise.

**Figure 4:**
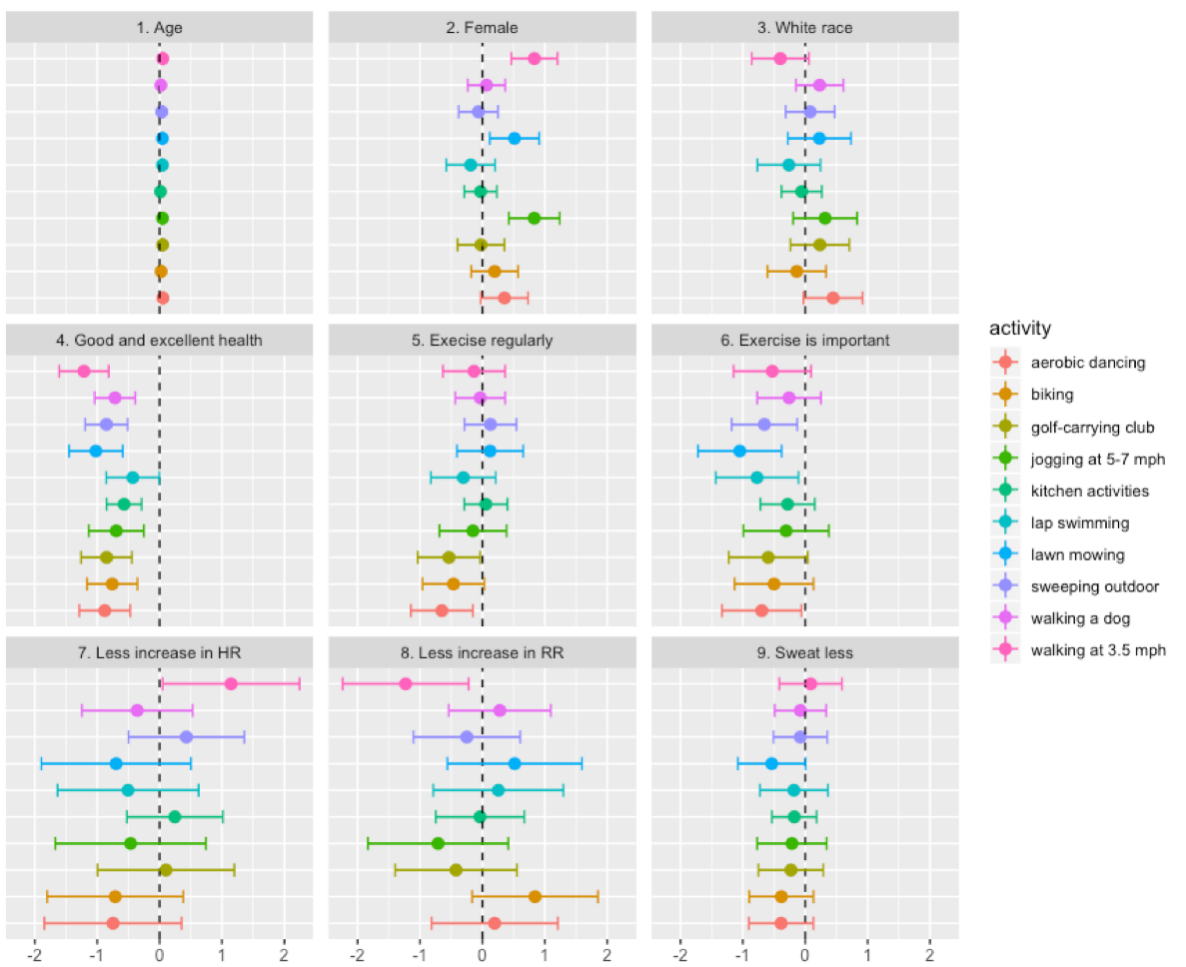
Regression coefficients and 95% CIs of the participants’ characteristics in predicting the intensity ratings of the 10 activity examples. HR: heart rate; RR: respiratory rate.

Figure 4 presents the coefficients of the 9 explanatory variables and their 95% CIs in predicting the numeric intensity ratings of the 10 activity examples. The regression analysis showed that age did not affect the intensity ratings at all. In addition, race, regular exercise, and physical exertion levels the respondents usually experienced after exercise were not strong predictors of the intensity ratings, except for a few activity types. For example, regular exercisers tended to rate lower the intensity of walking, golfing, and aerobic dancing than their counterparts. Overall, those who reported being in good health and considered regular exercise was important for their health tended to rate lower the intensity of the example activities. Female participants tended to rate higher the intensity of walking, walking a dog, lap swimming, and jogging than male participants.

## Discussion

### Principal Findings

The survey results showed that significant variations exist in how people perceive and express the intensity level of physical activity. Numeric rating of intensity with the perceived exertion level seemed to differentiate the 3 general intensity levels of mild, moderate, and vigorous relatively well. However, significant inconsistencies were observed in how the survey participants associated the descriptors commonly used in communicating exercise intensity with the intensity levels they perceived for various types of physical activity.

Physical exertion expressed with an increase in HR, breathing rate, and sweating did not always have a positive linear relation with exercise intensity. For example, some respondents indicated only a mild increase in these parameters after a moderate- or vigorous-intensity exercise, whereas others indicated a significant increase following a mild- or moderate-intensity exercise. This result underscores the importance of considering individual fitness levels and prior exercise habits when expressing activity intensity with these simplified degrees of physical exertion.

Providing specific activity examples is another popular method of describing activity intensity, as shown in many physical activity questionnaires. For example, jogging and aerobic exercise are often used as examples of moderate or vigorous activities, whereas walking is presented as an example of light- or moderate-intensity exercise. Although the intensity of certain activities was consistently rated higher than others in this survey, we also observed a wide variation in individual ratings given by the participants.

All the 10 activity examples have standardized MET values greater than 3, which indicates at least a moderate level of intensity. The participants gave different intensity ratings to the activities that shared the same standardized MET value. This finding confirms that standardized MET values are not a robust method for communicating activity intensities. Corrected MET values that incorporate one’s gender, age, and BMI can be an alternative that better quantifies the activity intensity at an individual level [12,28]. However, its usefulness as a means of representing and communicating personalized activity intensity should be further investigated.

Various characteristics of the respondents affected the intensity ratings of the 10 activity examples to varying degrees. The regression analysis showed that perceived health status and attitude toward regular exercise were stronger predictors of the intensity ratings of the example activities. Those who were in good health perceived presented activities less physically demanding, thus tended to rate lower than their counterparts. Similarly, those who responded that regular exercise is important for their health tended to rate the intensity of the presented activities significantly lower than their counterparts. The respondents’ demographics were not strong predictors of the numeric intensity rating, although the female respondents tended to rate jogging, swimming, and walking activities higher than the male respondents. In this survey, the race effect on the intensity rating was not apparent, except that the respondents with white race tended to rate walking higher than the respondents with other races.

According to the survey results, the majority of the participants who considered themselves in good health responded that they exercised regularly and that regular exercise was important to their health. One possible explanation for the significantly lower intensity ratings among the participants with good health is that, overall, they were in better physical fitness and thus usually experienced relatively less exertion from various physical activities. This finding suggests that physical fitness and exercise habit directly affect one’s intensity perception. Therefore, we may need to pay more attention to an individual person’s fitness and exercise habits when selecting physical activity examples to communicate activity intensity.

The survey results did not show any noticeable associations between the intensity level of exercise that the participants usually performed and the physical exertion they experienced after exercise measured with the simplified degrees of increase in HR, breathing rate, and sweating. Similarly, the relative exertion levels experienced after exercise were not among the participants’ characteristics most significantly associated with the different intensity ratings of the 10 activity examples. Those who reported experiencing relatively lower exertion after exercise tended to give lower intensity ratings than their counterparts for some activities, but this trend did not stand out compared with other participants’ characteristics. This finding suggests that describing activity intensity solely with the simplified degrees of exertion presented with the level of increase in HR, breathing rate, and sweating can be vulnerable to misinterpretation.

### Limitations

As per any study that involves survey data, this study is not free from data quality issues. As an attempt to include valid responses only, we removed the cases with a large percentage of missing or implausible answers (eg, giving an intensity rating of 10 to all 10 activity examples). However, there is no guarantee that the anonymized responses collected for this study are the truthful reflection of participants’ characteristics and their perceptions of physical activities.

Using the MTurk, we obtained study participants who were restricted to being enrolled in a crowdsourcing venue as a worker. The participants of such surveys may not represent the health status and behaviors of the general US population [26,29]. The sampled participants also comprised the majority (399/498, 79.9%) of white individuals. These 2 sample characteristics could limit the generalizability of the findings of this study.

### Practical Implications

Despite the limitations noted earlier, this study provides useful insights into communicating physical activity with patients. This study confirmed that wide variations exist in how people perceive and interpret the activity intensity expressed by general degree modifiers, physical exertion levels, and activity examples, which are the commonly used methods of describing physical activity intensity in everyday communication. The main lessons learned from this study are highlighted next.

First, this study showed the importance of considering individual differences in exercise habits and physical fitness when discussing physical activity with patients or assessing participants’ physical activity level in health behavioral studies. Second, the findings of this study indicate the need to adopt activity intensity descriptors that are easily implementable and sensitive to individual variations in intensity perception. For example, numeric intensity ratings seemed to provide a relatively reliable quantification of activity intensity that individual people experience. The talk test is another simple method for assessing and describing an individualized activity intensity level. Studies have reported mixed findings of their validity as a means of assessing physical exertion after exercising at a precise level [8,22,23]. However, they offer a quick and intuitive method for expressing a personalized intensity level of physical activity and thus can be considered as an alternative approach to describe activity intensity when communicating healthy lifestyle recommendations with patients. Mobile sensor devices may also provide a workable solution to this problem, given that the physical activity types, physical exertion level, and amount are accurately captured. Health behavioral studies that quantify participants’ physical activity may need to extend the use of mobile sensor devices to measure activity intensity.

### Conclusions

A survey of 498 adult volunteers showed that there exist statistically significant variations in how they perceived and interpreted the intensity of physical activity described using methods widely used in physical activity assessment and documentation. General degree modifiers, activity examples, and the simplified degree of physical exertion do not always convey accurate intensity information because of an individual’s internal calibration of the concept of activity intensity. The connection between quantitative standardized metrics and self-reported responses to clinically routine inquiry methods shows wide variations because of individual differences in one’s perception and interpretation of those intensity descriptions. If the purpose of assessing and documenting a patient’s physical activity level is simply to inquire whether a patient is physically active or not, scrutinizing the precise semantics of intensity concepts might not practically be a critical task. However, to provide clinically meaningful information, revisiting how we describe the intensity attribute of a patient’s physical activity seems necessary. We believe there is a need to consider an alternative approach that allows a more accurate and reliable characterization of the intensity level that an individual patient experiences with various physical activities.

## Appendix

Multimedia Appendix 1 The survey questionnaire implemented for this study.

## Abbreviations

%HR max: maximum heart rate
%VO2 max: maximum oxygen uptake rate
EHR: electronic health record
FITT: frequency, intensity, time, and type
HR: heart rate
MET: metabolic equivalent of the task
MTurk: Amazon Mechanical Turk
ONC: Office of National Coordinator for Health Information Technology
RPE: Rating of Perceived Exertion
