# Supplementary material for: Ambiguity in Communicating Intensity of Physical Activity: Survey Study
Source: JMIR Public Health Surveill. 2020 May 28;6(2):e16303. doi: 10.2196/16303 (PMC7290482; doi:10.2196/16303)
Supplement: Multimedia Appendix 1 [file publichealth_v6i2e16303_app1.pdf]

Exercise Intensity

Start of Block: Default Question Block

*

Q1 Your age in year:

____

Q2 Your gender:

- ◯ Male (1)
- ◯ Female (2)
- ◯ Other (3)

Q3 Your race/ethnicity:

- ◯ American Indian or Alaska Native (1)
- ◯ Asian (2)
- ◯ Black or African American (3)
- ◯ Hispanic or Latino (4)
- ◯ Native Hawaiian or Other Pacific Islander (5)
- ◯ White (6)
- ◯ Other (8)

Q6 How do you feel about your overall health?

- ◯ Poor (1)
- ◯ Fair (2)
- ◯ Good (3)
- ◯ Excellent (4)

Q7 Do you exercise regularly?

- ◯ Yes (39)
- ◯ No (40)

Q8 Do you have any chronic medical condition or an injury that affects your exercise?

- ◯ Yes (1)
- ◯ No (2)
- ◯ Not sure (3)

Q13 What is your usual exercise habit?

Frequency (1)

- Never
- 1-2 days/week
- 3-4 days/week
- 5-6 days/week
- everyday

Duration (2)

- Less than 30 min
- 30-60 min
- 60-90 min
- More than 90 min

Intensity (3)

- Mild
- Moderate
- Vigorous

Q16 Please think about your usual exercise. How do you usually feel after exercising?

	just a little (1)	moderately (2)	a lot (3)
I sweat (1)	○	○	○
My breathing rate increases (2)	○	○	○
my heart beat increases (3)	○	○	○

Q17 Again, please think about your usual exercise. What is the exertion level you usually feel after exercising? Please score the exertion level with a number between 1 to 20, 20 being extremely exerted

____

Q20 Please indicate how intense each of the following activities would be for you.

	Intensity level (1: very light, 10: extremely vigorous) 0 1 2 3 4 5 6 7 8 9 10	Not sure
Jogging at a pace of 5-7 miles per hour ()		
Walking at a pace of 3.5 miles per hour ()		
Golf - walking and carrying clubs ()		
Aerobic dancing (like Zumba) ()		
Fast lap swimming, freestyle ()		
Biking at a park ()		
Walking a dog ()		
Sweeping garage, sidewalk or outside of house ()		
Kitchen activities like cooking, washing dishes, cleaning up ()		
Lawn mowing with hand mower ()		

Q18 How important for you to be physically active or exercise regularly?

- ◯ Extremely important (1)
- ◯ Very important (2)
- ◯ Moderately important (3)
- ◯ Slightly important (4)
- ◯ Not at all important (5)

Q19 Do you monitor your physical activity level using an activity tracker such as fitbit and Apple watch?

○ Yes (1)

○ No (2)

End of Block: Default Question Block
